# Evaluation of risk factors for mortality and long-term survival after repair of acute type-A aortic dissection in 836 patients

**DOI:** 10.1186/1749-8090-8-S1-O43

**Published:** 2013-09-11

**Authors:** B Tutkun, S Buz, A Abd El Al, F Büttner, M Pasic, R Hammerschmidt, Y Weng, R Hetzer

**Affiliations:** 1 Cardiothoracic and Vascular Surgery, Deutsches Herzzentrum Berlin, Berlin, Germany

## Background

This study was designed to explore predictive factors for mortality and long-term survival in patients with acute type-A aortic dissection. We retrospectively assessed our data over a 15-year period starting in 1996.

## Methods

Between 01/1996 and 09/2011, 836 patients (559 men) with a mean age of 59.6±13.6 (range 18-92) years underwent surgery for acute type-A aortic dissection. No patients were excluded from immediate operation irrespective of age and preoperative status unless uncontrollable hemorrhage and/or cardiac arrest occurred before the patient reached the operating room. Ninety-two perioperative variables were statistically analyzed to identify predictors for early mortality.

## Results

The overall 30-day mortality was 22.3% (without cardiogenic shock 18.4%). The mortality rate was 9.8 % in patients aged < 45 years and 34.6 % in older patients aged ≥ 80 years. In the last 5 years the overall mortality was reduced to 17.7 % (without cardiogenic shock 15.3%). A multivariable logistic regression model showed that age >60 years (OR 1.03, 95%CI, 1.01 to 1.04, P<0.001), preoperative high inotropic score (OR 1.9, 95% CI 1.1 to 3.0, P<0.001), and additional CABG (OR 2.6, 95% CI 1.6 to 4.1, P<0.001) were predictors of 30-day mortality.

The long-term survival and freedom from reoperation at 1, 5 and 10 years were 70.5%, 58.5%, 42.1% and 96.4%, 91.2%, 88.8%, respectively.

## Conclusions

The results of this study support our institutional policy for acute type-A aortic dissection of not excluding any patients from the operation regardless of preoperative status and age.

